# Synaptic Vesicle Glycoprotein 2A Is Affected in the Central Nervous System of Mice with Huntington Disease and in the Brain of a Human with Huntington Disease Postmortem

**DOI:** 10.2967/jnumed.121.262709

**Published:** 2022-06

**Authors:** Daniele Bertoglio, Jeroen Verhaeghe, Leonie Wyffels, Alan Miranda, Sigrid Stroobants, Ladislav Mrzljak, Celia Dominguez, Mette Skinbjerg, Jonathan Bard, Longbin Liu, Ignacio Munoz-Sanjuan, Steven Staelens

**Affiliations:** 1 Molecular Imaging Center Antwerp (MICA), University of Antwerp, Wilrijk, Belgium;; 2 Department of Nuclear Medicine, Antwerp University Hospital, Edegem, Belgium; and; 3 CHDI Management/CHDI Foundation, Los Angeles, California

**Keywords:** SV2A, ^11^C-UCB-J, spinal cord, Huntington disease, animal model

## Abstract

Synaptic dysfunction is a primary mechanism underlying Huntington disease (HD) progression. This study investigated changes in synaptic vesicle glycoprotein 2A (SV2A) density by means of ^11^C-UCB-J small-animal PET imaging in the central nervous system of mice with HD. **Methods:** Dynamic ^11^C-UCB-J small-animal PET imaging was performed at clinically relevant disease stages (at 3, 7, 10, and 16 mo) in the heterozygous knock-in Q175DN mouse model of HD and wild-type littermates (16–18 mice per genotype and time point). Cerebral ^11^C-UCB-J analyses were performed to assess genotypic differences during presymptomatic (3 mo) and symptomatic (7–16 mo) disease stages. ^11^C-UCB-J binding in the spinal cord was quantified at 16 mo. ^3^H-UCB-J autoradiography and SV2A immunofluorescence were performed postmortem in mouse and human brain tissues. **Results:** ^11^C-UCB-J binding was lower in symptomatic heterozygous mice than in wild-type littermates in parallel with disease progression (7 and 10 mo: *P* < 0.01; 16 mo: *P* < 0.0001). Specific ^11^C-UCB-J binding was detectable in the spinal cord, with symptomatic heterozygous mice displaying a significant reduction (*P* < 0.0001). ^3^H-UCB-J autoradiography and SV2A immunofluorescence corroborated the in vivo measurements demonstrating lower SV2A in heterozygous mice (*P* < 0.05). Finally, preliminary analysis of SV2A in the human brain postmortem suggested lower SV2A in HD gene carriers than in controls without dementia. **Conclusion:** ^11^C-UCB-J PET detected SV2A deficits during symptomatic disease in heterozygous mice in both the brain and the spinal cord and therefore may be suitable as a novel marker of synaptic integrity widely distributed in the central nervous system. On clinical application, ^11^C-UCB-J PET imaging may have promise for SV2A measurement in patients with HD during disease progression and after disease-modifying therapeutic strategies.

## Visual Abstract

**Figure:**
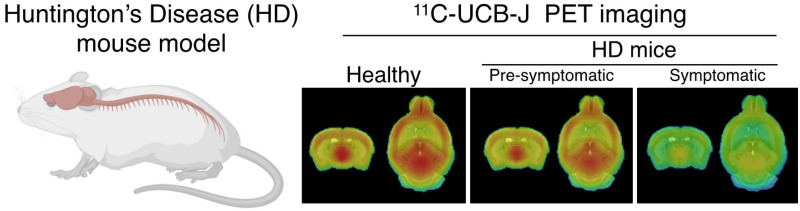

Huntington disease (HD) is an autosomal dominant neurodegenerative disorder caused by an expanded polyglutamine repeat in exon 1 of the gene encoding the huntingtin protein (*1*), leading to the expression of mutated huntingtin (mHTT). Pathologic features of HD include widespread progressive accumulation of mHTT, selective neurodegeneration, and forebrain atrophy (*2**,**3*).

A growing body of evidence suggests that mHTT induces synaptic transmission dysfunction (*4*), so synaptic dysfunction represents one of the main mechanisms underlying the progression of HD (*5*). Alterations in presynaptic proteins, including regulators of endocytosis and exocytosis of synaptic vesicles such as synaptosome-associated protein 25 and rabphilin 3A, have been reported in both clinical (*6**,**7*) and preclinical (*8*–*10*) postmortem studies. Previous work demonstrated that mHTT abnormally associates with synaptic vesicles, resulting in impaired synaptic function (*11*), and changes in synaptic proteins correlate with behavioral deficits (*10*), Thus, alterations in synaptic proteins may be suitable as candidate markers for monitoring HD progression (*12*–*14*). Given the current lack of effective treatment for preventing the disease or halting its progression, synaptic markers may play an important role in the development and evaluation of novel disease-modifying therapies throughout the entire central nervous system (CNS) (*15*).

Among presynaptic proteins, synaptic vesicle glycoprotein 2A (SV2A) is an essential vesicle membrane protein involved in neurotransmitter release and is expressed ubiquitously in synapses of the brain (*16**,**17*). Recent studies reported that SV2A can be imaged noninvasively in nonhuman primates, humans, and rodents by use of PET with the selective and high-affinity radioligand ^11^C-UCB-J (*18*–*20*). ^11^C-UCB-J PET may be suitable as a proxy for assessing synaptic density in vivo given its optimal clinical and preclinical pharmacokinetics and quantification properties (*20**,**21*). Thus, it provides a quantitative measure of synaptic changes during HD progression.

Here, we investigated ^11^C-UCB-J PET imaging for quantifying cerebral SV2A levels at clinically relevant disease stages in the knock-in Q175DN mouse model of HD (*22*–*24*). Additionally, given the evidence of mHTT pathology in the spinal cord (*25*), we evaluated the use of ^11^C-UCB-J PET imaging for detecting SV2A density changes in the rodent cervical spinal cord. Finally, postmortem measurements of SV2A were obtained in the mouse brain as well as in the human brain in a preliminary exploratory evaluation.

## MATERIALS AND METHODS

### Animals

Male wild-type (WT) mice (*n* = 35) and age-matched heterozygous knock-in Q175DN littermates (*n* = 35) (C57BL/6J background; CHDI-81003019) were obtained from Jackson Laboratories. The animals were housed singly in individually ventilated cages under a 12-h light/dark cycle in a temperature- and humidity-controlled environment with food and water ad libitum and at least 1 wk to acclimatize. All experiments were approved by the Ethical Committee for Animal Testing (ECD 2017–27) at the University of Antwerp (Antwerp, Belgium) and followed European Committee Guidelines (decree 2010/63/CEE).

The Q175DN model displays moderately slow disease progression with the hallmark of mHTT accumulation increasing from 3 to 12 mo (*26*). This animal model features motor deficits appearing around 6 mo and cognitive decline around 10 mo (*22**,**27*). Thus, ^11^C-UCB-J PET imaging was performed at clinically relevant disease stages: cross-sectional at 3 mo (presymptomatic stage; 16 mice per genotype); longitudinal at 7, 10, and 16 mo (appearance, progression, and advanced symptomatic stages, respectively; 19 mice per genotype).

### Tracer Radiosynthesis

^11^C-UCB-J synthesis was performed on an automated synthesis module (Carbosynthon I; Comecer) by adapting the previously described procedure (*18*) to our system (*20*). Average radiochemical purity was greater than 99%, and molar activity (mean ± SD) was 96.5 ± 13.3 GBq/μmol.

### ^11^C-UCB-J Dynamic Small-Animal PET Scan

Small-animal PET/CT imaging was performed on 2 Siemens Inveon PET/CT scanners (Siemens Preclinical Solutions). Animal preparation was performed as previously described (*20*). At the start of the dynamic small-animal PET scan, animals were injected via the tail vein with a bolus of ^11^C-UCB-J (5.4 ± 1.3 MBq) over a 12-s interval (1 mL/min) by use of an automated pump (Pump 11 Elite; Harvard Apparatus). The activity was injected in a trace dose, keeping the cold mass within 2.0 μg/kg across time points for consistency. Data were acquired in list-mode format. After the small-animal PET scan, a 10-min CT scan (80 kV; 500 μA) was performed for coregistration and attenuation correction. Detailed information on the scan parameters is reported in Supplemental Table 1 (supplemental materials are available at http://jnm.snmjournals.org). Published work from our group (*20*) was reanalyzed for blocking validation of ^11^C-UCB-J binding in the spinal cord. Blocking was achieved by pretreatment with levetiracetam injected intraperitoneally at either 50 (*n* = 4) or 200 (*n* = 4) mg/kg 30 min before radioligand delivery. Representative SUV images were generated on the basis of the interval from 10 to 90 min.

### Image Processing and Analysis

Acquired PET data were reconstructed into 33 frames of increasing length (12 × 10 s, 3 × 20 s, 3 × 30 s, 3 × 60 s, 3 × 150 s, and 9 × 300 s). For quantitative analysis, all images were reconstructed using a list-mode iterative reconstruction with spatially variant resolution modeling, 8 iterations, and 16 subsets of the 3-dimensional ordered-subset expectation maximization algorithm (*28*). Normalization, dead time, and CT-based attenuation corrections were applied. PET image frames were reconstructed on a 128 × 128 × 159 grid with 0.776 × 0.776 × 0.796 mm^3^ voxels. PET images were processed and analyzed using PMOD 3.6 software (PMOD Technologies).

Spatial normalization of the PET images was done through brain normalization of the PET images to an ^11^C-UCB-J PET template as previously described (*20*). Using the volume-of-interest template based on the Waxholm atlas (*29*), time–activity curves of different regions (striatum, motor cortex, hippocampus, and thalamus) were extracted from the images. The cervical spinal cord volume of interest was manually delineated on the individual CT images (by a researcher blind to condition), and time–activity curves were extracted. Kinetic modeling was performed to fit the time–activity curves to a standard 1-tissue compartmental model to determine the total volume of distribution (*V*_T_) by use of a noninvasive image-derived input function (IDIF); the *V*_T_ determined by use of the IDIF [*V*_T (IDIF)_] was used as a surrogate of the *V*_T_ estimate, as we recently validated (*20*). No genotypic difference in the plasma-to-whole blood ratio or plasma radiometabolites was present between genotypes; therefore, no correction was applied (*20*).

Parametric *V*_T (IDIF)_ and *K*_1_ maps were generated in PMOD through voxelwise analysis (1-tissue compartmental model) (*20*). Brain parametric maps were represented as averages for each genotype overlaid on a 3-dimensional mouse brain MRI template for anatomic reference, whereas maps focusing on the spinal cord were represented as data for an individual animal overlaid on CT.

### Mouse Brain Tissue

On termination of the longitudinal study, 16-mo-old animals (WT, *n* = 16; heterozygous, *n* = 13) were euthanized by decapitation while anesthetized, and brains were snap-frozen in 2-metylbuthane at −35°C for 2 min and preserved at −80°C until use. Serial sagittal sections (20 μm thick) were collected starting at 1.80 mm in the lateral bregma (*30*) in triplicate on Superfrost Plus slides (Thermo Fisher Scientific) using a cryostat (Leica).

### Postmortem Human Brain Tissue

Freshly frozen postmortem superior frontal gyrus tissue was obtained from The Netherlands Brain Bank, Netherlands Institute for Neuroscience (open access: www.brainbank.nl). All material was collected from donors for or from whom written informed consent for a brain autopsy and the use of the material and clinical information for research purposes had been obtained by The Netherlands Brain Bank. Ethics permission for the study was obtained from the Committee for Medical Ethics of the University of Antwerp/Antwerp University Hospital (20/13/155).

Tissue was obtained from female donors (age range, 50–67 y) with a postmortem interval of less than 8 h for all cases. Because SV2A has been reported to be decreased in patients with Alzheimer disease (*31*), tissue was assessed for evidence of neurologic morbidities (β-amyloid and tauopathy) through immunostaining. After the exclusion of controls who did not have dementia but were positive for β-amyloid aggregates or tau tangles and the exclusion of symptomatic HD gene carriers who were positive for β-amyloid aggregates or tau tangles, only 1 control without dementia (ID 2017–005; 60-y-old female; postmortem interval = 5.5 h) and 1 symptomatic HD gene carrier (ID 2017–060; 57-y-old female; postmortem interval = 6.7 h) were included in the investigation. Although the CAG repeat length for individuals with HD was not available in The Netherlands Brain Bank database, the presence of mHTT aggregates was confirmed histologically. Serial sections (10 μm thick) were collected on Superfrost Plus slides using a cryostat.

### Autoradiography

^3^H-UCB-J autoradiography was performed on mice at 16 mo as well as on postmortem human tissue as previously described (*32*) after the validation of SV2A selectivity using a blocking solution (1 nM ^3^H-UCB-J plus 1 mM levetiracetam in binding buffer) to validate ^3^H-UCB-J specificity for SV2A (Supplemental Fig. 1). ^3^H-UCB-J was synthesized at Pharmaron and had a molar radioactivity of 1,295 MBq/μmol and a radiochemical purity of greater than 99%.

Regional quantification was performed without knowledge of genotype using Fiji software (National Institutes of Health). ^3^H-UCB-J binding was measured in triplicate on 3 manually drawn slices. Region-specific binding of ^3^H-UCB-J was measured by converting the mean gray matter values into radioactivity density (Bq/mg) using commercial tritium standards (American Radiolabeled Chemicals). Next, using ^3^H-UCB-J molar activity on the day of the experiment, radioactivity density was converted into binding density (fmol/mg) for each region.

### Immunofluorescence

SV2A immunofluorescence was determined in mice at 16 mo as well as in postmortem human tissue. Sections were air dried for 5 min and incubated with 4% paraformaldehyde for 15 min for tissue postfixation. Next, slices were rinsed using phosphate-buffered saline (PBS), and nonspecific binding sites were blocked using 20% normal donkey serum in PBS for 1 h. Then, sections were incubated with an anti-SV2A primary antibody (rabbit antimouse; 1:400; 66724 [Cell Signaling Technologies]) in antibody diluent containing 5% normal donkey serum in PBS overnight at room temperature. On the next day, sections were washed with PBS before being incubated for 1 h with a secondary donkey antirabbit antibody (1:100; Alexa Fluor 488 [Jackson ImmunoResearch]) in antibody diluent containing 5% normal donkey serum in PBS. After washes with PBS, sections were mounted with 4′,6-diamidino-2-phenylindole (Vector Laboratories), and coverslips were added. Images at magnifications of ×20 and ×100 were acquired for quantification with a high-throughput fluorescence microscope (Nikon) with NIS-Elements Software (Nikon).

Quantification was performed without knowledge of genotype using Fiji software. Because the white matter was devoid of a specific signal, after conversion into an 8-bit gray scale, an intensity threshold was set to remove the background signal in the white matter (threshold, 27 of 255) and convert images into binary data. Regions of interest (striatum, motor cortex, hippocampus, and thalamus for mouse tissue; cortical gray matter for human tissue) were manually drawn on each image, and the percentage of surface area after thresholding was measured as the positive area. Quantification was done in triplicate (3 slices) for each region, and the average was used for statistical analysis.

### Statistical Analysis

All data were normally distributed, as assessed using the Shapiro–Wilk test. Longitudinal PET data were analyzed with a linear mixed model by fitting each region separately using ^11^C-UCB-J *V*_T (IDIF)_ or *K*_1_ determined by use of the IDIF as the dependent variable; genotype (WT and heterozygous), time (7, 10, and 16 mo), and the interaction between genotype and time (genotype × time) as fixed effects; and subjects as a random effect. The comparison was performed to evaluate regional temporal and genotypic differences. A 2-way ANOVA (with genotype and region as variables) was applied to investigate the 3-mo data and postmortem analyses. A 1-way ANOVA was used for blocking analysis in the spinal cord, whereas an unpaired *t* test was used to compare the genotypic differences in spinal cord SV2A PET. A Pearson correlation test was used to determine the relationship between variables. Normality and 2-way ANOVA were performed with GraphPad Prism (v 9.0) statistical software (GraphPad Software), analysis of the linear mixed model was performed with JMP Pro 13 (SAS Institute Inc.), and calculation of the effect size *d* was performed with G*Power software (http://www.gpower.hhu.de/). *P* values were corrected for multiple comparisons using the Tukey test. Data are represented as mean ± SD. All tests were 2-tailed, and statistical significance was set at *P* < 0.05.

## RESULTS

### SV2A Density Decreased with HD Progression

**FIGURE 1.**
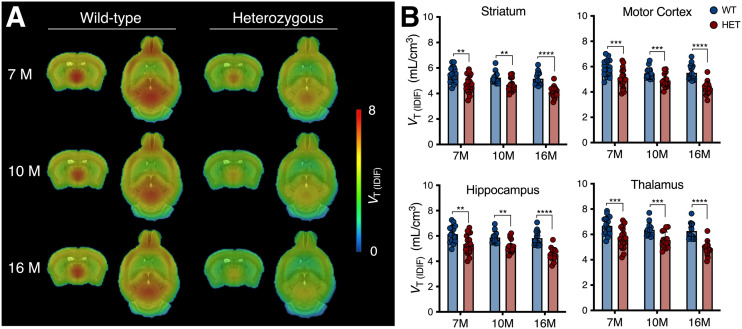
^11^C-UCB-J binding is reduced in symptomatic heterozygous (HET) mice. (A) Mean parametric ^11^C-UCB-J *V*_T (IDIF)_ maps of 7-, 10-, and 16-mo-old mice overlaid on MRI template for anatomic localization. (B) Cerebral *V*_T (IDIF)_ quantification denoting significant reduction in HET mice compared with WT littermates. **P < 0.01. ***P < 0.001. ****P < 0.0001.

Longitudinal mean *V*_T (IDIF)_ parametric maps of ^11^C-UCB-J at 7, 10, and 16 mo displayed a broad cerebral reduction of ^11^C-UCB-J binding in symptomatic heterozygous mice compared with WT littermates (Fig. 1A). Accordingly, ^11^C-UCB-J *V*_T (IDIF)_ values were significantly lower in heterozygous mice than in WT animals at all stages of disease investigated (i.e., 7, 10, and 16 mo) in parallel with HD progression (e.g., −13.4% ± 3.4% [*P* < 0.01], −10.8% ± 4.0% [*P* < 0.01], and −20.3% ± 4.0% [*P* < 0.0001] at 7, 10, and 16 mo, respectively, in the striatum) (Fig. 1B and Supplemental Table 2). Notably, the reduced ^11^C-UCB-J uptake was not related to altered *K*_1_ values (delivery rate of the tracer) (Supplemental Fig. 2), suggesting that the reduced binding did not reflect a mere decrease in cerebral perfusion.

**FIGURE 2.**
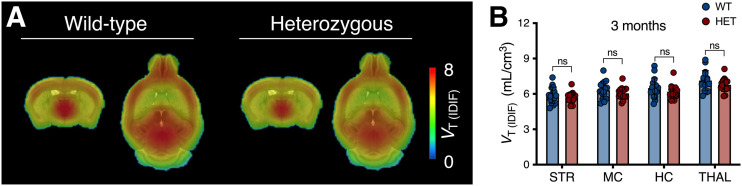
^11^C-UCB-J binding is not altered in presymptomatic heterozygous (HET) mice. (A) Mean parametric ^11^C-UCB-J *V*_T (IDIF)_ maps of 3-mo-old mice overlaid on MRI template for anatomic localization. (B) Cerebral *V*_T (IDIF)_ quantification at 3 mo does not differ between genotypes. HC = hippocampus; MC = motor cortex; ns = not significant; STR = striatum; THAL = thalamus.

No difference in ^11^C-UCB-J *V*_T (IDIF)_ values between WT and presymptomatic heterozygous mice (3 mo) was observed (*F*_1,116_ = 2.847 [*P* = 0.092]; e.g., −3.1% ± 4.1% for the striatum) (Fig. 2). *V*_T (IDIF)_ values at different ages are reported in Supplemental Table 3.

### SV2A Levels Were Reduced in Spinal Cord of Symptomatic Heterozygous Mice

**FIGURE 3.**
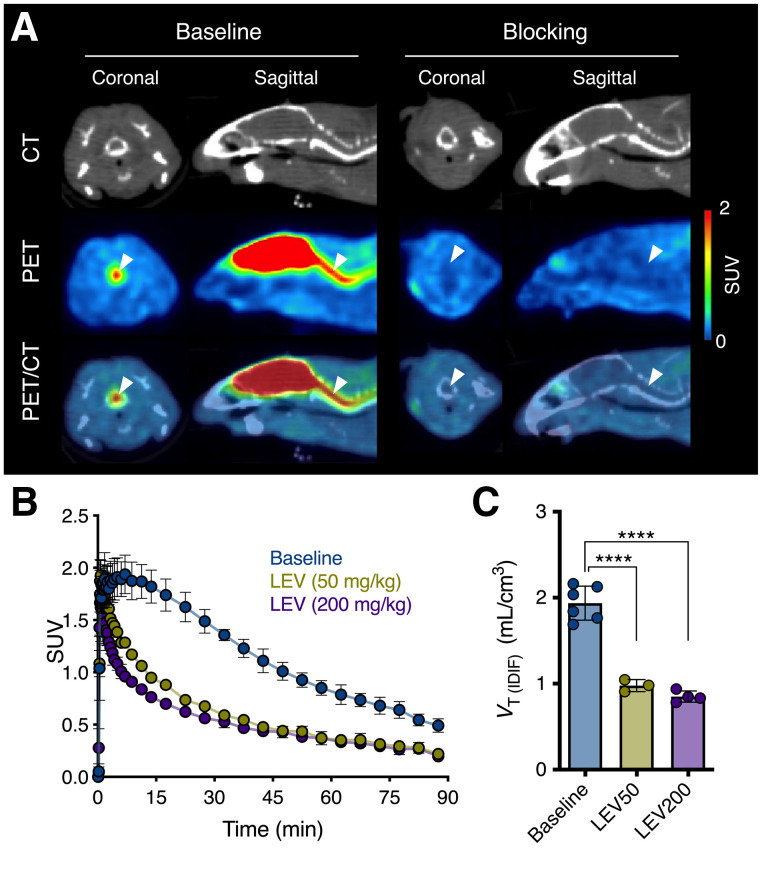
^11^C-UCB-J binding is specific and quantifiable in mouse spinal cord. (A) Representative ^11^C-UCB-J SUV PET/CT images of WT mouse at baseline and after pretreatment with levetiracetam (LEV, 200 mg/kg, intraperitoneal). Arrowheads indicate cervical spinal cord. (B) Cervical spinal cord SUV time–activity curves showing dose-dependent blocking effect. (C) Quantification of ^11^C-UCB-J *V*_T (IDIF)_ in cervical spinal cord. LEV50 = LEV at 50 mg/kg; LEV200 = LEV at 200 mg/kg. ****P < 0.0001.

We explored the potential application of ^11^C-UCB-J PET for the detection of SV2A in the mouse spinal cord. ^11^C-UCB-J binding was quantifiable and specific, as validated after pretreatment with levetiracetam (*F*_2,10_ = 78.96 [*P* < 0.0001]) (Fig. 3).

**FIGURE 4.**
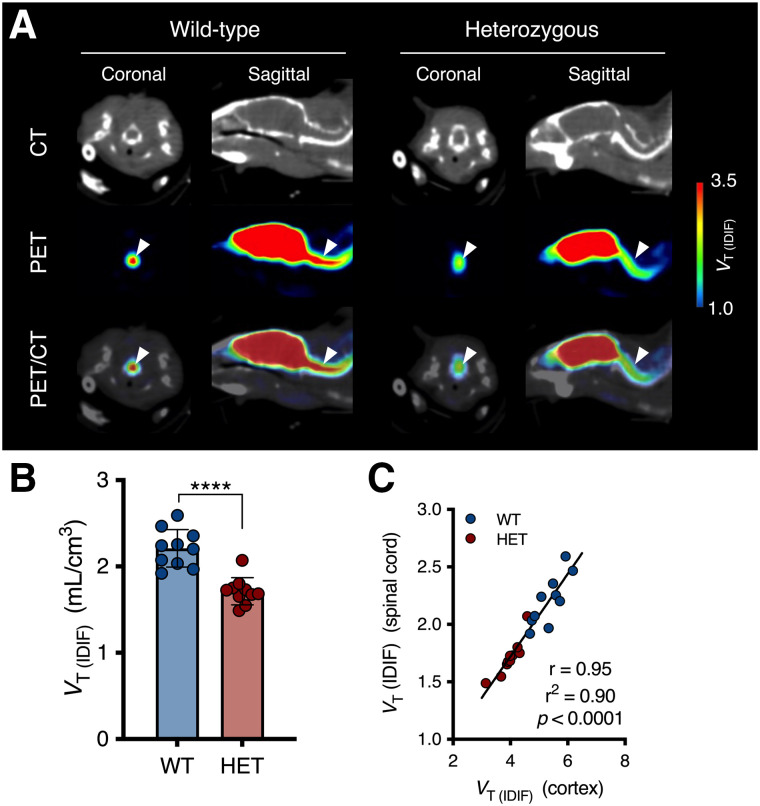
^11^C-UCB-J binding is decreased in spinal cord of symptomatic heterozygous (HET) mice. (A) Representative maps of 16-mo-old mice overlaid on CT. Arrowheads indicate cervical spinal cord. (B) Spinal *V*_T (IDIF)_ is significantly lower in 16-mo-old HET mice than in WT mice. ****P < 0.0001. (C) Correlation between spinal and cortical ^11^C-UCB-J binding.

Next, on the basis of clinical evidence indicating the presence of mHTT pathology in the spinal cord, we quantified ^11^C-UCB-J PET in the cervical spinal cord of symptomatic heterozygous mice (16 mo). ^11^C-UCB-J binding was significantly lower in the cervical spinal cord of heterozygous mice than in that of WT littermates (−22.5% ± 3.8% [*P* < 0.0001]) (Fig. 4B), and there was a strong association with cortical quantification (*r*^2^ = 0.90 [*P* < 0.0001]) (Fig. 4C).

### Postmortem Rodent and Human Studies Corroborated SV2A Reduction in HD

**FIGURE 5.**
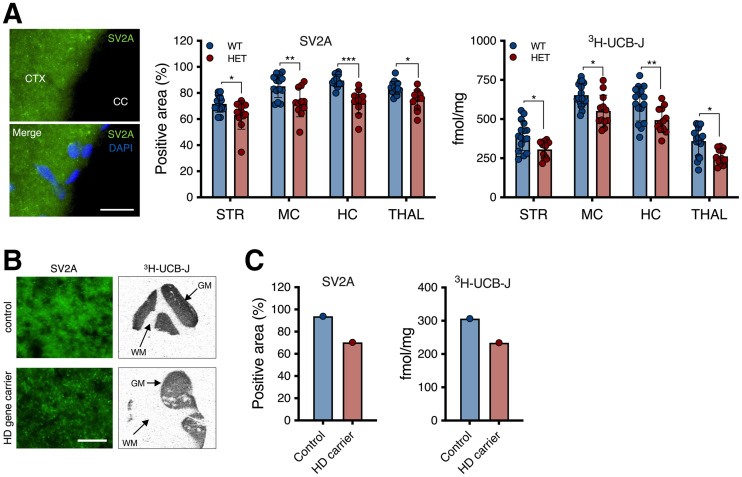
Postmortem quantification displayed SV2A reduction in HD. (A) SV2A immunostaining and ^3^H-UCB-J–specific binding in murine brain. (B) Representative SV2A immunostaining and ^3^H-UCB-J total binding autoradiograms of human control and HD gene carrier. (C) ^3^H-UCB-J–specific binding and SV2A immunostaining tissue suggest reduction in cortical SV2A in human tissue. *P < 0.05. **P < 0.01. ***P < 0.001. CC = corpus callosum; CTX = cortex; DAPI = 4′,6-diamidino-2-phenylindole; GM = gray matter; HC = hippocampus; HET = heterozygous; MC = motor cortex; ns = not significant; STR = striatum; THAL = thalamus; WM = white matter. Scale bar = 20 μm.

^3^H-UCB-J–specific binding was significantly lower in heterozygous mice than in WT littermates (*F*_1,104_ = 35.77 [*P* < 0.0001]; e.g., −22.1% ± 8.3% for the striatum), in agreement with the in vivo measurement and as corroborated by SV2A immunostaining (*F*_1,104_ = 51.42 [*P* < 0.0001]; e.g., −12.0% ± 4.5% for the striatum) (Fig. 5A).

We performed a preliminary assessment of ^3^H-UCB-J binding in postmortem human cortex tissues from a control and an HD gene carrier (Fig. 5B). Both ^3^H-UCB-J–specific binding and SV2A immunostaining indicated a lower SV2A signal in the HD gene carrier (Fig. 5C).

## DISCUSSION

This work assessed synaptic integrity using the PET radioligand ^11^C-UCB-J in heterozygous mice at clinically relevant presymptomatic and symptomatic stages of the disease. To our knowledge, this work represents the first evidence of in vivo changes in SV2A density. In particular, changes in synaptic density were detectable at all symptomatic stages of HD with mHTT accumulation broadly affecting SV2A levels in the entire CNS.

Despite mounting evidence indicating that mHTT induces presynaptic transmission dysfunction during the progression of HD (*4**,**5*), to date no clinical or preclinical studies have assessed alterations in presynaptic proteins in vivo. Nonetheless, cross-sectional findings in animal models of HD suggested a reduction in different synaptic proteins in different animal models at symptomatic but not presymptomatic stages of the disease (*8*–*10*), in agreement with our observations in vivo in heterozygous mice as well as in vitro in the tissues of mice with HD and postmortem human tissue.

Since the development of SV2A radioligands for in vivo imaging of SV2A (*18**,**19*), preclinical and clinical investigations have been restricted to the brain despite the fact that SV2A is distributed in all gray matter, including the spinal cord (*33*). Thus, on the basis of our previous levetiracetam blocking study (*20*), we evaluated the specificity of the ^11^C-UCB-J signal in the cortical spinal cord; we demonstrated the capability of SV2A PET imaging in the spinal cord of a living animal. Interestingly, Lambeng et al. reported a 2- to 3-fold difference in SV2A expression in the spinal cord compared with the cerebral cortex in the rat (*33*). In the present work, we measured a 2.5-fold difference in ^11^C-UCB-J binding between the spinal cord and the motor cortex, in agreement with the previous report for the rat (*33*). Next, on the basis of the clinical evidence of mHTT pathology in the spinal cord (*25*), we explored ^11^C-UCB-J binding in the cervical spinal cord of symptomatic heterozygous mice and observed a decline in SV2A density of a magnitude similar to that in the brain. Altogether, these observations support the exploration of SV2A PET imaging as a synaptic integrity marker in spinal cord–related disorders, such as amyotrophic lateral sclerosis, as well as spinal cord injury. On the basis of recent evidence that levetiracetam treatment leads to functional recovery in spinal cord injury models (*34*), the latter is currently being investigated by our group.

In recent years, the use of PET imaging in the identification of different striatal markers for monitoring HD progression has significantly progressed (*35*–*41*). However, since the whole CNS is affected in HD, noninvasive markers with a ubiquitous brain distribution, such as ^11^C-UCB-J PET imaging, may provide unique insights for elucidating global pathophysiologic changes during HD. Intriguingly, the clinical feasibility of detecting SV2A decline has been reported in other neurodegenerative disorders, including Alzheimer disease and Parkinson disease (*31**,**42*).

## CONCLUSION

Collectively, these findings demonstrate significant SV2A deficits in the brain and spinal cord of symptomatic heterozygous mice. ^11^C-UCB-J PET imaging may be a promising marker for the assessment of synaptic integrity in patients with HD during disease progression and after disease-modifying therapeutic strategies.

## DISCLOSURE

This work was funded by CHDI Foundation, Inc., a nonprofit biomedical research organization exclusively dedicated to developing therapeutics that will substantially improve the lives of HD-affected individuals. D. Bertoglio is supported by the Research Foundation Flanders (FWO; 1229721N) and the University of Antwerp (BOF KP; FFB210050). The University of Antwerp also funded the work through partial assistant professor positions for J. Verhaeghe and L. Wyffels and full professor positions for S. Stroobants and S. Staelens. L. Wyffels and S. Stroobants are also supported by Antwerp University Hospital through departmental positions. No other potential conflict of interest relevant to this article was reported.

## Notes

KEY POINTS **QUESTION:** Is SV2A density affected during the progression of HD? **PERTINENT FINDINGS:** In this ^11^C-UCB-J PET study, we demonstrated brain and spinal cord SV2A deficits during symptomatic HD in mice, highlighting the potential of SV2A PET as a marker in the entire CNS. **IMPLICATIONS FOR PATIENT CARE:** ^11^C-UCB-J PET imaging may be a unique CNS functional marker for HD and may have promise as an application for SV2A measurement in patients with HD during disease progression and following therapeutic interventions.
